# Performance Enhancing Effect of Metabolic Pre-conditioning on Upper-Body Strength-Endurance Exercise

**DOI:** 10.3389/fphys.2018.00963

**Published:** 2018-07-20

**Authors:** Philipp Birnbaumer, Alexander Müller, Gerhard Tschakert, Matteo C. Sattler, Peter Hofmann

**Affiliations:** ^1^Institute of Sports Science, Exercise Physiology, Training and Training Therapy Research Group, University of Graz, Graz, Austria; ^2^Department of Physical Activity and Public Health, Institute of Sports Science, University of Graz, Graz, Austria

**Keywords:** inhibition of glycolysis, pull-up exercise, lactate, metabolic pre-conditioning, lactate shuttle theory

## Abstract

High systemic blood lactate (La) was shown to inhibit glycolysis and to increase oxidative metabolism in subsequent anaerobic exercise. Aim of this study was to examine the effect of a metabolic pre-conditioning (MPC) on net La increase and performance in subsequent pull-up exercise (PU). Nine trained students (age: 25.1 ± 1.9 years; BMI: 21.7 ± 1.4) performed PU on a horizontal bar with legs placed on a box (angular hanging) either without or with MPC in a randomized order. MPC was a 26.6 ± 2 s all out shuttle run. Each trial started with a 15-min warm-up phase. Time between MPC and PU was 8 min. Heart rate (HR) and gas exchange measures (VO_2_, VCO_2_, and VE) were monitored, La and glucose were measured at specific time points. Gas exchange measures were compared by area under the curve (AUC). In PU without MPC, La increased from 1.24 ± 0.4 to 6.4 ± 1.4 mmol⋅l^−1^, whereas with MPC, PU started at 9.28 ± 1.98 mmol⋅l^−1^ La which increased to 10.89 ± 2.13 mmol⋅l^−1^. With MPC, net La accumulation was significantly reduced by 75.5% but performance was significantly increased by 1 rep (4%). Likewise, net oxygen uptake VO_2_ (50% AUC), pulmonary ventilation (VE) (34% AUC), and carbon dioxide VCO_2_ production (26% AUC) were significantly increased during PU but respiratory exchange ratio (RER) was significantly blunted during work and recovery. MPC inhibited glycolysis and increased oxidative metabolism and performance in subsequent anaerobic upper-body strength-endurance exercise.

## Introduction

The knowledge about the effects of Lactate (La) on performance and exercise metabolism has grown strongly during the past years. Former seen as the main factor in muscular fatigue and acidosis related tissue damage, La turned out to be an important product and regulator of exercise metabolism, which is produced even under fully oxidative conditions (Brooks, 2000, 2009). The interaction between La producing and La consuming muscle cells and their interaction with the body system can be explained by the lactate shuttle theory (Brooks, 1985, 2007, 2009). As soon, as exercise intensity increases, the intracellular La concentration starts to increase and La exchange along a gradient out of the cell is favored immediately as long as the systemic La level is low. The mechanisms of the “lactate shuttle" enable the removal of La from the muscle to the whole body system via monocarboxylate transporters (MCT’s) (Halestrep, 2013). At low exercise intensity, La is oxidized by mitochondria of the muscle cell, which initially produced La (intracellular lactate shuttle), or is delivered from a glycolytic cell to an oxidative cell without entering the bloodstream (Cell to Cell shuttle). With increasing exercise intensity, La production exceeds the intramuscular shuttle capacity and La enters the blood stream where it is mainly removed and combusted by oxidative (muscle) fibers in the active muscle bed, the heart and other tissue. At very high intensities, La production exceeds the limits of whole body La oxidation and increases accumulatively. Aerobic and anaerobic energy supply is therefore based on La transport mechanisms like MCT’s along proton and concentration (Brooks, 2009; Halestrep, 2013). This relationship has been shown for incremental (Hofmann and Tschakert, 2011) as well as for constant load (Tschakert and Hofmann, 2013; Moser et al., 2017) and interval type exercise (Moser et al., 2015; Tschakert et al., 2015).

La concentration affects performance in multiple ways during exercise and warm-up. As already known, warm-up exercise is a generally accepted practice to improve supra-maximal performance (Robergs et al., 1991; Gray and Nimmo, 2001). Reasons for the performance increasing effects of active warm-up, like increased body- and muscle temperature and the activation of the aerobic metabolism have already been described (McGowan et al., 2015) but are still discussed (Beckenkamp and Lin, 2011). High intensity warm-up compared to moderate and low intensity warm-up leads to a significantly higher oxygen uptake and lower net La concentration at the same performance outcome in subsequent high intensity exercise (Wittekind and Beneke, 2011; Anderson et al., 2014). These studies suggest that pre elevated systemic La concentration influences performance and exercise metabolism in multiple ways. However, pre-conditioning exercise may play a crucial role in this relationship. Bailey et al. (2009) examined the effect of systemic pre-conditioning on oxygen uptake kinetics and exercise tolerance in subsequent severe cycle ergometer exercise. Participants performed different combinations of prior exercise intensity, recovery time, and cycling intensity. Prior exercise at 70% of the difference between the work rates at the gas exchange threshold (GET) and the maximal oxygen uptake (VO_2max_) led to increased oxygen kinetics in all tests and increased exercise tolerance by 30% with 20 min of recovery, respectively, 15% with 9 min of recovery. Several other studies showed that prior intense exercise, respectively, priming exercise boosts VO_2_ kinetics and increases oxygen uptake and performance (DiMenna et al., 2008; Bailey et al., 2009, 2016; Barker et al., 2010). However, adding a specific priming exercise may offer performance-enhancing options by influencing oxygen kinetics and metabolism.

Parolin et al. (1999) investigated the effect of repeated maximal exercise on subsequent high intensity exercise bouts, which provides insight into the nature of priming exercise. The key finding was that La and pyruvate concentration in bout 3 remained nearly constant at the level of the final concentration of bout one. The prior exercise bouts increased oxidative metabolism along with a decrease in anaerobic glycolysis and a reduction in exercise performance and net La increase. Similar effects were shown when La was pre-elevated by an intense warm-up, La increase was significantly reduced and mean power remained unaffected (Wittekind and Beneke, 2011).

Bogdanis et al. (1994) already showed that the rate of La accumulation after cycle ergometer sprints was decreased by approximately 50% when La was already pre-elevated by hand crank exercise. In a recent study, which was largely based on the experimental design of Bogdanis et al. (1994), Grant et al. (2014) also showed a decrease in La accumulation in 30 s maximal Wingate sprints after La concentration was pre-elevated by intense arm exercise (biceps curl). Although La increase was significantly blunted, there was no effect on performance outcomes in this study. The reduction of La accumulation in response to exercise was suggested from a reduced anaerobic contribution. This reduction after prior high-intensity exercise likely reflects a reduced anaerobic glycolytic energy contribution, as suggested by several studies (Klausen et al., 1972; Gerbino et al., 1996; Fukuba et al., 2002; Carter et al., 2005; Wittekind and Beneke, 2011).

Furthermore, pre-conditioning may also alter performance outcomes. Already Klausen et al. (1972) examined the effect of pre-elevated high La concentrations on maximal exercise performance. Participants performed a supra-maximal leg cycle ergometer test until complete exhaustion with and without a prior 5 min heavy arm exercise. Results showed a significant reduction in net La increase, a consistent VO_2max_, a slightly higher ventilation (VE) and a not statistically significant 10% reduction in performance. The authors concluded that a high initial La concentration does not affect aerobic power output, which is in contrast to the common understanding of the effects of high systemic La concentrations on performance. Müller et al. (2013), who investigated the effect of a prior high intensity anaerobic arm exercise on subsequent high intensity anaerobic leg-cycling exercise also showed a significant reduction of net La increase by 50% with the same exercise intensity and duration. Prior anaerobic exercise with the upper-body induced a shift in the metabolic contribution from an anaerobic to a more oxidative metabolism in the subsequent anaerobic leg exercise. These authors suggested beneficial effects in sports with sub-maximal anaerobic energy contribution, when inducing pre-elevated La concentrations by non-sport specific muscle groups. Recently, Purge et al. (2017) investigated the effect of a high systemic La concentration, induced by a prior non-rowing-specific arm crank exercise but with a sport specific muscle group, on subsequent all-out 2000-m rowing exercise. After a 25 s high intensity anaerobic upper-body all-out arm crank exercise, rowing performance was reduced by 1% with substantially reduced glycolytic energy contribution. Elevated systemic La concentrations therefore seem to regulate exercise metabolism depending on La flux rate and direction (Müller et al., 2013; Purge et al., 2017).

The aim of this study was to investigate the effects of a high systemic La concentration, induced by high-intensity leg exercise, on subsequent net La increase and performance in dynamic pull-up exercise (PU). It was hypothesized that systemic La elevation by high intensity leg exercise reduces La accumulation, while there is no change in performance in subsequent PU.

## Materials and Methods

### Participants

Nine male university students (age: 25.1 ± 1.9 years; height: 181.6 ± 4.9 cm; weight: 71.8 ± 7.7 kg; BMI: 21.7 ± 1.4) volunteered to participate in the study. They had been informed about the experimental procedures and possible risks and signed a written consent form. The study procedures have been approved by the local Research Ethics Committee. All participants were healthy and well trained, five were ambitious sports climber (climbing UIAA grade 7–9), four were recreationally active (e.g., Yoga, fitness-training, and soccer). The study specific PU was not familiar to all participants and the procedure was explained at the start of the study. On test days, subjects were instructed to visit the laboratory in a rested state, without strenuous exercise within the previous 24 h. Finally, there have been no dropouts or missing data in the present study.

### Experimental Design

**FIGURE 1:**
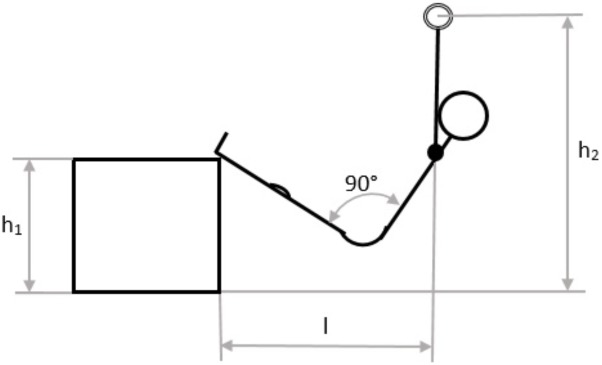
Schematic test setting.

All participants visited the laboratory twice with at least 2, maximum 7 and mean 3.4 ± 2 days in between. Each participant completed a test under two conditions: with and without metabolic pre-conditioning (MPC). The order of the conditions was randomized. In the test phase, the participants had to perform PU with the target to accomplish as many repetitions as possible. PU was performed on a horizontal bar with legs placed on a box to reduce pull-up intensity and create a strength endurance load comparable to that in sports climbing (Booth et al., 1999; Sheel, 2004; Watts, 2004). The experimental settings were customized for each participant depending on anthropometric measures. The setting was defined in the first visit, protocolled and marked with tape. In all further tests, the defined mark positions were used. The main factors of positioning were: center off mass underneath the horizontal bar, shoulder-wide grip and stretched legs with heels on the box and approximately 90° hip flexion (**Figure 1**). Participants were instructed to do shoulder width pull-up’s in full range of motion with a constant movement velocity in which every repetition cycle lasted about 2 s, 1 s for the concentric and 1 s for the eccentric phase. The maximum number of repetitions until failure was counted and participants were given strong verbal encouragement throughout each test. When participants could not maintain the defined constant velocity or were no longer able to perform a full range of motion PU although verbally encouraged was defined as exhaustion point. MPC was an all-out shuttle run in order to increase La to about 8 mmol·l^−1^. Participants were told to run repeatedly three stages (6, 13.5, and 21 min) as fast as possible for a period of about 25 s. MPC was performed 5 min after a standardized warm-up and was followed by an 8 min seated MPC-recovery phase. Based on the experience from previous studies (Müller et al., 2013; Purge et al., 2017) a MPC La concentration of about 8 mmol·l^−1^ was suggested to be an optimum, choosing a shuttle run setup increasing La to approximately that value. All participants started PU 29 min after the start in non-MPC and MPC. In the standardized warm-up, participants performed 10 min of cycling at 70 W at 70 RPM (MONARK ERGOMEDIC 839 E, Monark, Sweden) followed by 10 squat jumps while slow-running, mobilization exercise for shoulder, wrist and ankle as well as 6 pull-up’s in the experimental high bar setup (**Figure 1**).

### Measurements

**FIGURE 2:**
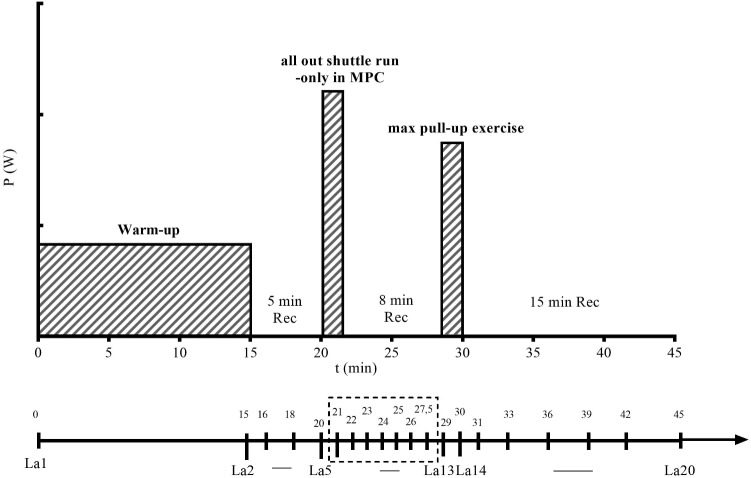
Exercise testing protocol for the maximal pull-up exercise (PU) with metabolic pre-conditioning (MPC). *X*-axis indicates time in min (0–45) when blood samples (La1–La20) were collected to measure lactate concentration (La). *Y*-axis indicates the intensity of warm-up, MPC, and PU in Watt (W). In non-MPC, time points were the same except for the MPC phase and the samples La5–La13, which were not collected.

Participant’s body height and body mass were measured and BMI was calculated. To determine blood-lactate and glucose concentrations, blood samples (20 μl) were collected from the earlobe in both tests at similar time points. In non-MPC, blood samples were taken before and immediately after the warm-up procedure, in minute 1, 3, and 5 after the warm-up, at the start and immediately after PU. Final samples were taken in minute 1 and 3 of recovery and every third min of the following 12 min. In MPC, the structure and time points were the same, except for the all-out shuttle run where blood samples were taken before (5 min after warm-up), after MPC, every minute during the first 5 min of MPC-recovery and after 6.5 min of MPC-recovery (**Figure 2**). La and glucose concentrations were determined with an enzymatic-amperometric method (Biosen S_line, EKF-Diagnostics GmbH, Germany). During exercise testing, subject’s heart rate (HR) was measured continuously by a HR monitor (Polar S810i, Kempele, Finland) and recorded in 5 s interval. Gas exchange data such as oxygen consumption (VO_2_), carbon dioxide production (VCO_2_), and minute VE, were continuously measured in breath-by-breath mode by a portable gas analyzer (CORTEX METAMAX 3B, Cortex Biophysik GmbH, Germany). Gas sensors and volume calibration were performed before each test according to the manufacturer’s guidelines. VO_2_ uptake kinetics were determined as 50% net VO_2_ increase during PU and time to reach 50% net VO_2peak._ After PU participants were instructed to rate their perceived exertion (RPE) after every 7th PU and at maximum.

### Statistical Analyses

To determine the overall effect of time, condition, and time × condition on net La, we applied two linear mixed-effects models for longitudinal data with subjects defining the second level of analysis using Stata 12 (Stata-Corp, College Station, TX, United States). In the first model, the following variables were included: time, condition, and time × condition. In the main model, we included all variables of the first model, and additionally, the quadratic effects of time (i.e., time^2^, condition × time^2^). For both models, we used restricted maximum likelihood (REML) estimates and mean centered the variable time (i.e., before calculating interaction and quadratic effects). We considered the inclusion of quadratic effects as highly relevant in order to model the curvilinear course of net La.

Net increase in La and gas exchange variables were calculated by subtraction of the value before PU (time point La13, **Figure 2**) from the values measured in PU and recovery. Any other analyses were performed using Graph Pad Prism 5 (Graph Pad Software, United States). To determine differences at follow up measurements, we performed paired *t*-tests in case of normal distribution, and otherwise, Wilcoxon tests. Normal distribution was assessed by Shapiro–Wilk, Q-Q-Plots, and Histogram. Finally, the area under curve (AUC) was calculated for La, VO_2_, VCO_2,_ and VE during PU. The results are expressed as means ± SD and means with 95% confidence intervals for La production and net increase. The level of significance was set at *p* ≤ 0.05 for the overall analysis and at *p* ≤ 0.01 for all pairwise-comparisons.

## Results

### Performance

**FIGURE 3:**
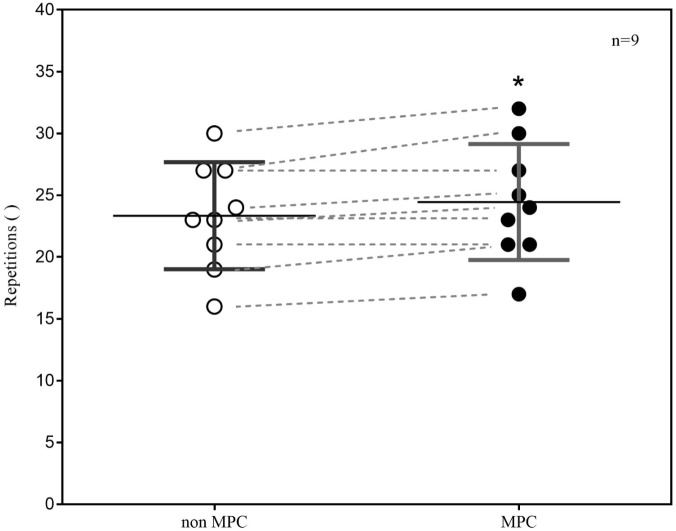
Pull-up exercise (PU) performance (repetitions) in non-MPC and MPC (means ± SD, pre–post comparison). **^∗^***p* = 0.01.

Metabolic pre-conditioning significantly affected the mean number of accomplished pull-up’s in the test (non-MPC: 23.33 ± 4.08 Rep; MPC: 24.44 ± 4.42 Rep; *p* ≤ 0.01). This increase of one repetition is equal to a performance increase of 4%. Within comparison of performance outcome shows, that performance did not decrease in MPC in any case but increased in six and remained the same in three participants (**Figure 3**).

### Blood Lactate, Glucose, and Gas Exchange Kinetics

**FIGURE 4:**
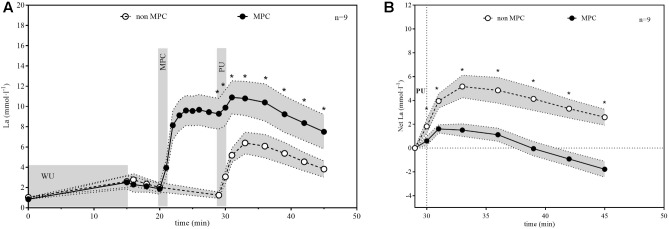
**(A)** Lactate (La) concentration in non-MPC and MPC (means ± 95%CI). **(B)** Net La increase during pull-up exercise (PU) and recovery (means ± 95%CI). MPC increases La to high levels followed by a reduced La increase in PU and recovery compared to no MPC. Net La increase was significant reduced in the MPC (^∗^*p* < 0.01).

The La responses in non-MPC and MPC are illustrated in **Figure 4**. We increased the systemic La concentration by anaerobic shuttle runs to a maximum value of 9.67 ± 1.97 mmol·l^−1^ which resulted in a mean La concentration of 9.28 ± 1.98 mmol·l^−1^ at the start of PU. La concentrations reached similar values after the standardized warm-up (VO_2mean_ during warm-up: 1.52 ± 0.43, respectively, 1.47 ± 0.4 l min^−1^) in both tests (non-MPC 2.6 ± 0.76, MPC 2.5 ± 0.83 mmol⋅l^−1^). In MPC, La concentration increased rapidly with the all-out shuttle run (26.6 ± 2 s) whereas in non-MPC La decreased between warm-up and PU to almost resting values. PU caused a comparatively steep La increase in non-MPC, whereas in MPC La kinetics were clearly blunted (**Figure 4A**). **Figure 4B** shows the net La increase during PU (net La increase in non-MPC compared to MPC 5.16 ± 1.24 vs. 1.61 ± 0.45 mmol⋅l^−1^) and recovery. In the first model (χ^2^ = 344.5, *p* < 0.001; level 2: *SD* = 0.73, 95% CI [0.41, 1.29]), there was a non-significant effect of time (*b* = −0.0008, SE = 0.07, 95% CI [−0.14, 0.14], *p* = 0.99), a significant effect of condition (*b* = −3.39, SE = 0.20, 95% CI [−3.78, −3.00], *p* < 0.001) as well as a significant interaction between time and condition (*b* = −0.49, SE = 0.10, 95% CI [−0.68, −0.30], *p* < 0.001). In the main model (χ^2^ = 1195.2, *p* < 0.001; level 2: *SD* = 0.77, 95% CI [0.46, 1.29]), there were significant effects for condition (*b* = −3.89, SE = 0.18, 95% CI [−4.24, −3.54], *p* < 0.001), condition × time (*b* = −0.49, SE = 0.06, 95% CI [−0.61, −0.38], *p* < 0.001), time^2^ (*b* = −0.30, SE = 0.02, 95% CI [−0.35, −0.25], *p* < 0.001) and condition × time^2^ (*b* = 0.12, SE = 0.03, 95% CI [0.06, 0.19], *p* < 0.001), whereas the effect of time (*b* = −0.0008, SE = 0.04, 95% CI [−0.08, 0.08], *p* = 0.98) was not significant.

**FIGURE 5:**
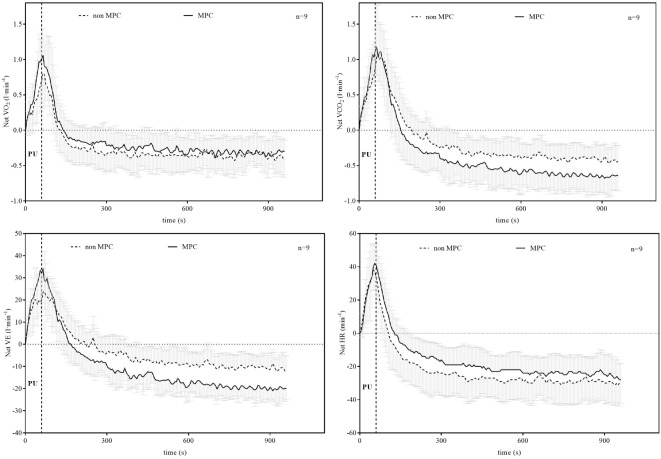
Net oxygen uptake (VO_2_), net carbon dioxide output (VCO_2_), net ventilation (VE), and net heart rate (HR) during pull-up exercise (PU) and recovery with and without MPC (means ± SD). Net values were calculated by subtracting the value before PU from the values measured during PU and recovery.

**FIGURE 6:**
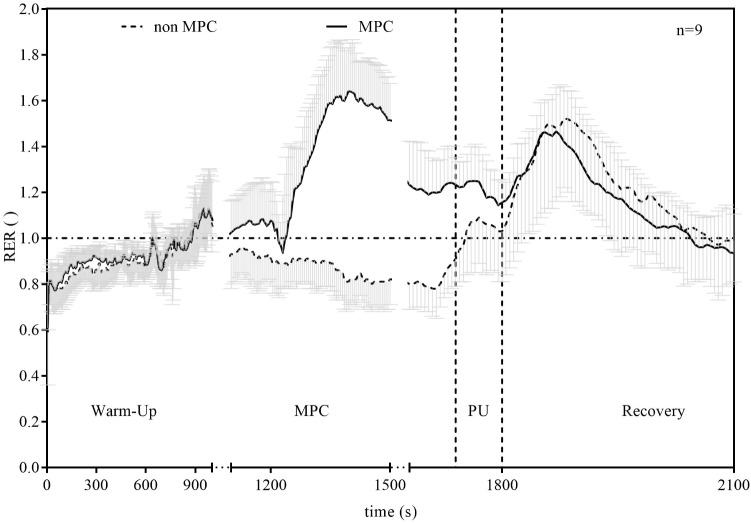
Respiratory exchange ratio (RER) in non-MPC and MPC (means ± SD). *X*-axis is plotted in three segments whereby parts between warm-up and MPC as well as between MPC and PU where cut for clarity.

Significant pairwise comparisons are shown in **Figure 4**. Finally, AUC indicated that there was 75.5% less net La increase in MPC compared to non-MPC. No significant differences were found for blood glucose values at rest (4.74 ± 0.62 vs. 4.86 ± 0.4 mmol⋅l^−1^), the start of PU (4.74 ± 0.81 vs. 5.15 ± 0.61 mmol⋅l^−1^) and for peak value after PU (4.89 ± 0.68 vs. 5.36 ± 0.73 mmol⋅l^−1^) between control and MPC. The spirometric variables showed significant increases in net oxygen uptake (50% AUC), net carbon dioxide output (26% AUC) and VE (34% AUC) during PU in MPC (**Figure 5**). The peak values for VO_2_, VCO_2_, and VE, during PU are significantly greater in MPC compared to non-MPC (**Table 1**). VO_2_ uptake at the start of PU was not significantly different between conditions (**Table 1**). 50%-VO_2peak_ was significantly higher in MPC compared to non-MPC (0.46 ± 0.22 vs. 0.68 ± 0.19 l min^−1^), but time at 50%-VO_2peak_ was not significantly different between conditions (35.58 s vs. 36.47 s). In addition, resting, warm-up and values before MPC were not significantly different between both conditions. In MPC, VCO_2_ values were still elevated at the onset of PU and remained higher compared to non-MPC during PU and recovery. The illustration of net VCO_2_ (**Figure 5**) shows significantly higher mean values during PU in MPC compared to non-MPC but a much faster net VCO_2_ decline to the initial value, similar as VO_2_ and VE (**Figure 5**) which was also significantly increased in MPC compared to non-MPC. Respiratory exchange ratio (RER) increased during MPC exercise to high values, which decreased in MPC-recovery phase and increased only slightly at the start of PU. In contrast, in non-MPC RER increased substantially with the start of PU and reached higher values during the recovery phase. In both conditions, there was a decrease of RER toward the end of PU (**Figure 6**). Finally, all gas exchange variables indicated higher oxygen uptake and oxidative metabolism after high-intensity MPC.

**Table 1:** Pull-up exercise (PU) pre and peak values in non-MPC and MPC of heart rate, gas exchange data, and perceived exertion.

	non-MPC	MPC
HR_pre_ (bpm)	115 ± 12.91	124 ± 15.69
HR_peak_ (bpm)	153 ± 20.54	166 ± 19.55^∗^
VO_2*pre*_ (l·min^−1^)	0.82 ± 25	0.82 ± 20
VO_2peak_ (l·min^−1^)	1.62 ± 0.48	1.88 ± 0.37^∗^
VCO_2*pre*_ (l·min^−1^)	0.74 ± 0.22	1.01 ± 0.21^∗^
VCO_2peak_ (l·min^−1^)	1.80 ± 0.78	2.19 ± 0.46^∗^
VE_pre_ (l·min^−1^)	25.62 ± 7.16	36.97 ± 4.91^∗^
VE_peak_ (l·min^−1^)	49.41 ± 18.63	71.20 ± 13.93^∗^
RPE_25/50/75/100_	9.1/12.6/16.1/18.9	8.5/12.4/16.3/19.1

*^∗^Different from non-MPC (p < 0.05). Values are means ± SD. HR_pre_, VO_2pre_, VCO_2pre_, and VE_pre_ denote heart rate, oxygen uptake, carbon dioxide output, and ventilation immediately before PU. HR_peak_, VO_2peak_, VCO_2peak_, and VE_peak_ denote the peak values of the measured physiological parameters. RPE_25/50/75/100_ denote the rated perceived exertion at 25, 50, 75, and 100% of maximum PU. MPC denotes metabolic pre-conditioning (MPC) by anaerobic leg exercise.*

## Discussion

The major findings of this study were that prior high-intensity anaerobic exercise with non-specific muscles significantly reduced net La increase by 75.5% but significantly increased PU performance by 4%. Although the mean performance increase was only one repetition a clear trend to a higher performance outcome after MPC was seen as performance increased in six and remained the same in three participants. The results support a performance enhancing potential of high systemic La concentrations on subsequent PU.

The reduced net La increase can be explained by the lactate shuttle theory (Brooks, 1985, 2007, 2009). For example, our results are in line with findings of other studies performing anaerobic exercise prior to a high-intensity exercise in order to induce an elevated systemic La concentration at the onset of exercise (Klausen et al., 1972; Bogdanis et al., 1994; Müller et al., 2013; Grant et al., 2014; Purge et al., 2017). These studies showed a reduction in net La increase between approximately 44 and 53%, when La was pre-elevated by anaerobic arm exercise and followed by a high-intensity leg exercise. The greater reduction in net La increase in our study can be explained by a quantitative difference in La production of the smaller arm muscles compared to the leg muscles (Wirtz et al., 2014). Glucose consumption was not significantly different between conditions and unaffected by MPC. Oxygen uptake, carbon dioxide output, VE, and RER were significantly higher during PU in MPC compared to non-MPC. RER was higher during PU in MPC compared to non-MPC due to a higher starting value induced by MPC exercise, but decreased during PU. The smaller increase and rapid return to baseline levels of RER during recovery in MPC can be explained by a reduced local acidosis due to a reduced net La production, respectively, the reduced H^+^ ions emission (Wasserman et al., 1973). This was already shown in previous studies, investigating the metabolic effects of high intensity intermitted exercise as well as of different warm-up strategies on performance (Parolin et al., 1999; Wittekind and Beneke, 2011; Grant et al., 2014). Contrary to the net La accumulation, the parameters of VE increased substantially in MPC. Oxygen uptake was accelerated and the net increase was up to 50% higher in MPC compared to non-MPC during PU (**Figure 5**). This accelerated VO_2_ kinetic in high-intensity exercise is a well-known phenomenon after priming exercise (DiMenna et al., 2008; Bailey et al., 2009; Barker et al., 2010). VO_2_ kinetics were significantly faster in MPC in line with previous results (DiMenna et al., 2008; Bailey et al., 2009; Barker et al., 2010). This priming effect inducing a faster and higher oxygen uptake consequently leads to reduced anaerobic contribution with the same work intensity. On the other hand, inhibition of glycolysis due to MPC is suggested to force a higher oxidative contribution such as shown in earlier results (Parolin et al., 1999). From our data, we cannot conclude either hypothesis, but due to the markedly stronger net lactate reduction (Müller et al., 2013; Purge et al., 2017) compared to the smaller VO_2_ increase, we favor the inhibition hypothesis, although recognizing a priming effect also.

Barker et al. (2010) showed that priming exercise resulted in faster overall VO_2_ kinetics and elevated muscle oxygenation prior to and throughout a subsequent exercise, although phase II VO_2_ kinetics were unaltered. They suggested that the availability of muscle O_2_ at the onset of exercise does not limit the initial increase in VO_2_. DiMenna et al. (2008) found similar results and suggested, that VO_2_ kinetic relate to metabolic properties of the muscle fibers recruited. As we may suggest that then inhibition of La is primarily limiting fast glycolytic fibers we assume, that oxygen uptake during PU in MPC is favored to compensate for the decreased ability to produce La, which could be confirmed by the VO_2_ and VCO_2_ kinetics during PU and in the recovery phase in our study. This is in line with findings from Burnley et al. (2002), who showed that an increased primary VO_2_ amplitude after prior high intensity exercise was related to an additional motor unit recruitment. This higher recruitment in the second bout of heavy cycling exercise was suggested to compensate fatigued fibers to maintain power output. The increased number of recruited motor units would lead to reduced tension per fiber, increased O_2_ cost of exercise but reduced muscular metabolic disturbance (Burnley et al., 2002). This could explain the somehow paradox effect of a lower lactate increase but unchanged VCO_2_ (**Figure 5**) output in MPC. Contrary to Burnley et al. (2002), however, an additional motor unit recruitment in our study is not caused by muscular fatigue, but we suggest a decreased ability of fast fiber for anaerobic metabolism inhibited by high systemic lactate concentration. Anyhow, we suggest an inhibited anaerobic glycolysis during PU due to high La concentrations induced by a prior anaerobic leg exercise, which inverts the La gradient enhancing oxidative metabolism.

Correia-Oliveira et al. (2017) showed similar effects, lowering blood pH by oral ammonium chloride intake, which induced acidosis and caused a significant reduction in power output and anaerobic expenditure rate during a 4 km cycling time trail. They assumed that a reduced H^+^ gradient across the sarcolemma, caused by an increase of extracellular H^+^, reduces monocarboxylate transporter activity and the efflux of H^+^ from the muscle to the blood. This may inhibit muscle glycogenolytic and glycolytic flux caused by lower intramuscular pH during exercise. Our present study showed a reduced La gradient due to high systemic lactate concentration in MPC accompanied by a reduced net La increase, indicating an inhibition of glycolysis. This effect was markedly stronger compared to the acidosis caused by oral intake of ammonium chloride in the study by Correia-Oliveira et al. (2017).

A key factor in our study was the La concentration at the onset of exercise. According to other studies, which showed that La concentrations between 8 and 10 mmol·l^−1^ were effective to influence subsequent high intensity exercise (Bogdanis et al., 1994; Müller et al., 2013), we increased the systemic La concentration by anaerobic shuttle run’s to a maximum value of 9.67 ± 1.97 mmol·l^−1^. Our prior exercise resulted in a mean La concentration of 9.28 ± 1.98 mmol·l^−1^ at the beginning of PU in MPC. The increase in performance was independent of La concentration after MPC. However, a too high MPC exercise intensity may cause excessively high systemic disturbances and therefore decrease performance, whereas a too low intensity will not affect performance, which was already shown in studies examining the effects of warm-up intensity (Wittekind and Beneke, 2011; Anderson et al., 2014). Bailey et al. (2009) described the effect of a sufficiently intense prior high intensity exercise and a sufficiently long recovery duration for homeostasis to return toward control level on performance. In our study, the recovery time after MPC was 8 min. La accumulation reached its maximum 5 min after MPC and was already lower at the end of MPC-recovery phase. In addition, VO_2_, VCO_2_, VE, and HR were declining toward base levels at the end of MPC-recovery phase in order to keep cardiorespiratory strain at a minimum, despite high La levels. MPC (26.6 ± 2 s all out shuttle run) in combination with 8 min of recovery increased systemic La to high levels and ensured sufficient regeneration of all subjects.

As recently shown for rowing, (Purge et al., 2017) it may be difficult to apply such a MPC approach for whole body exercises. MPC may be useful in strength endurance sports like sports climbing. For example, Watts et al. (1996) found a significant correlation between post-climbing La concentration and decreases in handgrip strength and showed that La increased from 1.4 ± 0.8 mmol·l^−1^ to 6.1 ± 1.4 mmol·l^−1^ when climbing a difficult route. Therefore, a shift to an increased oxidative metabolism in the working muscle could postpone failure caused by muscle metabolic fatigue. This is supported by recent studies, who showed that intracellular acidosis was responsible for muscle fatigue and performance limitations (Volianitis et al., 2010). If net La increase is reduced due to high systemic La concentration, a reduced intracellular La production along with a reduced intracellular acidosis may be a beneficial result. However, contrary to the study of Purge et al. (2017), the active muscles in MPC exercise were not substantially involved in PU in our study, which may explain the difference in effects.

Further potential of a systematic inhibition of net La increase can be suspected in training situations. Several studies demonstrated the inhibition of glycolysis during repeated intervals in high intensity interval training (HIIE) (Parolin et al., 1999; Tschakert et al., 2015). Likewise, our study showed that the effect of a forced dominant oxidative metabolism under an anaerobic training condition, like in HIIE, can also be archived by a high systemic La level induced by none dominant working muscles in a MPC exercise bout. Further, these findings open new perspectives in training as well as training-methods and present a possibility to induce oxidative working conditions already at the start of high intensity exercise by manipulating the systemic La concentration.

### Limitations

As we did not measure biopsies we cannot prescribe the molecular mechanism occurring during such a MPC exercise bout but we may suggest that at least some of the effects may be prescribed like in the Parolin study (Parolin et al., 1999). Their study showed that glycogen utilization was markedly reduced down to negligible from the first to the third bout of maximal intermittent exercise. These authors suggested La accumulation appeared due to an imbalance of the relative activities of glycogen phosphorylase (Phos) and pyruvate dehydrogenase (PDH). The increase in H^+^ concentration reduces pyruvate production by inhibiting Phos transformation and simultaneously activate PDH in the third bout such that there is a better matching between pyruvate production and oxidation with minimal lactate accumulation. As each bout progressed and with successive bouts, there was a decreasing ability to stimulate substrate phosphorylation through phosphocreatine hydrolysis and glycolysis and a shift toward greater reliance on oxidative phosphorylation (Parolin et al., 1999). An additional reason for the increase in oxidative metabolism and performance could be an intramuscular switch from mainly fast to slow twitch fibers which could explain the increased oxygen uptake but not the increase in performance such as shown by Sundberg et al. (2017). These authors found an increased EMG activity due to the recruitment of additional motor units and a change in discharge rate of the active motor units as a compensatory mechanism used by the nervous system to maintain a constant force output as the muscle fibers became fatigued (Sundberg et al., 2017). A further limitation may be the fact that compensatory changes in intermuscular coordination may be expected which could not be controlled in the applied experimental setting. Cote et al. (2008) nicely prescribed that fatigue influenced motion at both local and global levels and specifically, interjoint and intermuscular coordination adapted to compensate for local effects of fatigue to maintain movement characteristics.

## Conclusion

Our study shows that a high systemic lactate concentration induced by exercise with unspecific muscles influenced local muscle metabolism and forced the muscle to an increased oxidative energy contribution. Although this effect has been already prescribed, we could show for the first time that this approach is also able to improve exercise performance. Understanding the specific details of the lactate shuttle theory (Brooks, 2018) allows therefore to manipulate metabolism offering some potential also regarding exercise training, sports competition, and health (Hofmann, 2018).

## Ethics Statement

This study was carried out in accordance with the recommendations of the Helsinki Declaration. The protocol was approved by the Ethics committee of the University of Graz. All subjects gave written informed consent in accordance with the Declaration of Helsinki.

## Author Contributions

PH, PB, and AM conceived and designed the experimental plan. PB and AM performed the experiments. PB and MS analyzed the data. PB and PH drafted the manuscript. GT, PH, and MS refined and approved the final manuscript. All authors proofread and accepted the final version of the manuscript.

## Conflict of Interest Statement

The authors declare that the research was conducted in the absence of any commercial or financial relationships that could be construed as a potential conflict of interest.
